# Study on Aberrant Eating Behaviors, Food Intolerance, and Stereotyped Behaviors in Autism Spectrum Disorder

**DOI:** 10.3389/fpsyt.2020.493695

**Published:** 2020-11-05

**Authors:** Chunyan Li, Yang Liu, Hui Fang, Yu Chen, Jiao Weng, Mengyao Zhai, Ting Xiao, Xiaoyan Ke

**Affiliations:** ^1^Children's Mental Health Research Center, The Affiliated Brain Hospital of Nanjing Medical University, Nanjing, China; ^2^State Key Laboratory of Microbial Metabolism, School of Life Sciences and Biotechnology, Shanghai Jiao Tong University, Shanghai, China

**Keywords:** autism spectrum disorder, aberrant eating behaviors, gastrointestinal symptoms, food intolerance, stereotyped behaviors

## Abstract

**Objective:** To investigate the aberrant eating behaviors (EBs), gastrointestinal (GI) symptoms, and food intolerance in children with autism spectrum disorder (ASD) and their association with clinical core symptoms of ASD.

**Method:** A total of 94 preschool children with ASD treated at the Child Mental Health Research Center of Nanjing Brain Hospital between October 2016 and April 2018 were enrolled. In addition, 90 children with typical development (TD) in the community during the same period were recruited. The conditions of aberrant EBs and GI symptoms in children were investigated using questionnaire surveys. Serum specific IgG antibodies against 14 kinds of food were detected using enzyme-linked immunosorbent assays (ELISAs).

**Results:** The detection rate of aberrant EBs in the ASD group was significantly higher than that in the TD group (67.39 vs. 34.94%), and the rate of GI symptoms was also higher in the ASD group than that in the TD group (80.22 vs. 42.11%). Detection of food intolerance in children with ASD showed that the positive rate was 89.89% and that the majority of children had multiple food intolerances. The correlation analysis results showed that the severity of aberrant EBs positively correlated with stereotyped behavior of children with ASD (*r* = 0.21, *P* = 0.04) and that food-specific IgG antibodies concentrations positively correlated with high-level stereotyped behavior in children with ASD (*r* = 0.23, *P* = 0.03).

**Conclusion:** ASD with aberrant EBs or high food-specific IgG antibodies concentrations had more severe stereotyped behavior, which may have implications for exploring the immune mechanism of ASD.

**Clinical Trial Registration**: ChiCTR-RPC-16008139.

## Introduction

It has been more than 70 years since the American child psychiatrist Leo Kanner first formally reported and described infantile autism in 1943 (1). The medical community has seen many changes in the understanding of autism and its related disorders in the past 7 decades. Furthermore, there have been profound impacts on the clinical diagnosis and treatment of autism spectrum disorder (ASD). ASD is currently considered a group of highly heterogeneous neurodevelopmental disorders. In addition to 2 major core symptoms, social communication disorders and stereotyped and repetitive interests and behaviors, there are also many accompanied symptoms such as aberrant eating behaviors (EBs), gastrointestinal (GI) symptoms, food intolerance, sleep disorder, epilepsy, and attention deficit hyperactivity disorder. These accompanied symptoms not only increase difficulties in the clinical diagnosis of ASD but also increase the complexity of ASD treatment. The association between these accompanied symptoms, such as aberrant EBs, GI symptoms, and food intolerance, and ASD clinical core symptoms are still unclear. It is worthy of further investigation to determine whether these accompanied symptoms are present randomly, whether they are closely associated with core injuries of ASD, whether they are parts of ASD symptoms, or whether they participate in the pathogenesis of ASD. After a review of previous study reports, we found that, on one hand, there were large differences among all study reports, for example, the incidence of GI symptoms among the ASD population ranged from 4.2 to 96.8% (2)and the incidence of aberrant EBs ranged from 27 to 89% (3, 4), on the other hand, the associations among these accompanied symptoms and their correlations with ASD clinical core symptoms were also inconsistent. For example, Yang et al., in 2017, showed that the incidence of GI symptoms among 169 children with ASD aged between 3 and 12 years was 47.93% and that children with GI symptoms had significantly increased scores on the stereotyped behavior submodule of the Autism Diagnostic Interview-Revised (ADI-R) and communication submodule of the Autism Diagnostic Observation Scale (ADOS) (5). In addition, the study by Prosperity et al. in 2017 of 163 preschool children with ASD showed that children with ASD with accompanied GI symptoms or aberrant EBs had more severe emotional problems or stereotyped behaviors than those without accompanying GI symptoms or aberrant EBs (4). In 2013, however, Chandler surveyed GI symptoms in 132 children with ASD aged between 10 and 14 years and showed that although the incidence rate of GI symptoms in children with ASD was 46.5% and significantly increased compared to that in the normal adolescent population, GI symptoms did not correlate with the severity of ASD clinical symptoms (6).

Children with ASD often show intolerance to one or more food. Food proteins and protein-derived compounds may modulate the immune response in the body. Leaky gut may lead to food-borne components into blood circulation, and resulting in an abnormal immune response and increased levels of circulating pro-inflammatory cytokines (7). Detection of food intolerance usually by test food-specific IgG antibodies. Previous study showed that Gluten and casein intolerance in children with ASD often have high-level specific IgG antibodies (8). Immune abnormalities may explain part of the pathogenesis of ASD.

For neurodevelopmental disorders that arise in the early stage of childhood and last for a lifetime, it is unclear which age groups with accompanying symptoms such as aberrant EBs, GI symptoms, and food intolerance should be focused on and which associations are worth addressing. The study by Brensnahan showed that the incidence rates of GI symptoms in children with ASD aged between 6–18 months and between 18–36 months were both significantly higher than that in typical development children (9). Some researchers even suggested listing feeding difficulty in infants as a symptom in the early screening of ASD (10, 11). To our knowledge, few studies have combined EBs, GI symptoms and IgG antibodies of food intolerance in ASD, and explore their relationship to ASD. Therefore, the present study surveyed aberrant EBs, GI symptoms, and food intolerance in preschool children with ASD in East China and further investigated the association among these accompanying symptoms and their correlation with ASD clinical core symptoms.

## Materials and Methods

### Participants

#### Patient Group

Patients were children with ASD treated at the Children's Mental Health Center of Nanjing Brain Hospital between October 2016 and April 2018. The following were the inclusion criteria: patients who conformed to the ASD diagnostic criteria in the Diagnostic and Statistical Manual of Mental Disorders, 5th Edition (DSM-5); two senior child psychiatrists performed diagnosis based on disease history, clinical evaluation, and diagnostic criteria, and patients who had inconsistent diagnostic results were excluded; patients who were aged between 2 and 6 years; patients who lived in Nanjing and surrounding areas for more than 5 years (or lived in Nanjing or surrounding areas since birth); and patients who had permission from guardians to participate in this research. The exclusion criteria were as follows: patients who had genetic or metabolic diseases such as Rett's syndrome or fragile X syndrome; patients who had a clear history of craniocerebral trauma; and patients who had a history of nervous system diseases and severe somatic diseases. A total of 94 cases of young children with ASD aged between 25 and 82 months were enrolled in this study. The average age was (47.97 ± 13.82) months. There were 78 boys and 16 girls, and the boys to girls' ratio was 4.88:1.

#### Control Group

Children with typical development (TD) in the community during the same period were recruited. The inclusion criteria were as follows: ①children age and gender matched with the ASD group; ②children aged between 2 and 6 years; ③children who lived in Nanjing and surrounding areas for more than 5 years (or lived in Nanjing or surrounding areas since birth); and ④children who had permission from guardians to participate in this research. The exclusion criteria were as follows: ①children who had various neurodevelopmental disorders and mental disorders; ②children who had a clear history of craniocerebral trauma; and ③children who had a history of nervous system diseases and severe somatic diseases. A total of 90 cases of TD children aged between 24 and 79 months were enrolled. The average age was (51.35 ± 10.65) months. There were 73 boys and 17 girls. The boys to girls' ratio was 4.29:1.

This research was reviewed and approved by the Medical Ethics Committee of the Affiliated Brain Hospital of Nanjing Medical University (approval number: 2016–KY017). Written informed consent from all guardians were obtained, and the informed consent includes clinical blood collection.

### Measures

#### Clinical Assessment

General demographic data, previous disease history, personal history, aberrant EBs, and GI symptoms of all enrolled children were surveyed using the self-complied general condition questionnaire. The aberrant EBs and GI symptoms involved in this questionnaire were common symptoms in ASD children selected based on clinical observation and relevant studies. The characteristics and severity of clinical symptoms of children with ASD were evaluated using the ADI-R, ADOS, Childhood Autism Rating Scale (CARS), and Autism Behavior Checklist (ABC). For the ADI-R, the scores for 5 submodules were calculated in our study, social interaction, stereotyped behavior, high-level stereotyped behavior, low-level stereotyped behavior, and disease development, were calculated. The sum of C1 and C2 in stereotyped behavior was recorded as high-level stereotyped behavior, and the sum of C3 and C4 was recorded as low-level stereotyped behavior (12). The ADOS scale was divided into 5 submodules, communication, social interaction, combined, imagination/creativity, and stereotyped behave/restricted interests. Each module had the corresponding cut-off score. The total score was calculated for the CARS scale, and the cut-off score was 30 points. The ABC scale included scores for 5 factors, body and object use (B), language (L), sensory (S), relating (R), and social and self-help (SC), and the cut-off score was 57 points. When the scores for the above scales were higher, the ASD symptoms were more severe. The Gesell Developmental Schedules (Gesell) was used to evaluate development level. This scale includes 5 domains: adaption, gross motor, fine motor, language, and social development. The developmental quotient (DQ) of each domain >85 was considered normal, <75 was considered abnormal, and between 75 and 85 was considered suspicious.

#### Food Intolerance Detection

Specimen collection and processing: After patients were enrolled, 2 mL of peripheral venous blood was collected using an EDTA-K2 anticoagulant tube, and the blood was shaken and centrifuged (3,000 r/min for 10 min). The supernatant was collected and stored in a −80°C freezer. Specimens were detected in batches. Detection method: The concentrations of food-specific IgG antibodies were detected using enzyme-linked immunosorbent assays (ELISAs) in an Anthos Lucy 2 ELISA reader (Austria). The reagent kits for the detection of 14 types of food-specific IgG antibodies were purchased (HOB Biotech, Jiangsu) to test the concentration of specific IgG antibodies for each food. The detection results were divided into 4 levels: negative (–, <50 U/ml), mild intolerance (+, 50~100 U/ml), moderate intolerance (++, 100~200 U/ml), and severe intolerance (+++, >200 U/ml). The positive items for food intolerance of all individuals divided by the total items was recorded as the positive rate.

### Statistical Analysis

Data analysis was performed using SPSS 23.0 software. Measurement data are expressed as the mean ± standard deviation (*x* ± *S*). The differences in severity of GI symptoms and EB symptoms between the ASD group and the TD group were compared using the independent sample *t*-test. The detection rates of GI symptoms and aberrant EBs between the ASD group and the TD group were compared using the χ^2^-test. The association between GI symptoms, EB symptom, and IgG antibody concentrations as well as ASD clinical symptoms was analyzed using Pearson correlation analysis. *P* < 0.05 indicated a significant difference.

## Results

### Comparison of General Conditions Between the ASD Group and the TD Group

The differences in gender composition (χ^2^ = 0.11, *P* = 0.74), age (*t* = −1.84, *P* = 0.07), and body mass index (BMI) (*t* = 0.29, *P* = 0.77) of the participants in these two groups were not significantly different. DQ values of the 5 domains of the Gesell in the ASD group were all significantly lower than those in the TD group; the difference was significant (*P* < 0.001) (Table 1).

**Table 1:** Comparison of the general conditions of the children in the 2 groups.

	**ASD group**	**TD group**	***t/χ*^**2**^**	***P-*value**
Age (months)	47.97 ± 13.82	51.35 ± 10.65	−1.84	0.07
Gender (*n*)	–	–	0.11	0.74
Boy	78	73		
Girl	16	17		
Delivery method	–	–	4.72	0.30^*^
Natural	38	48		
Cesarean section	46	29		
BMI	16.36 ± 6.54	16.15 ± 1.87	0.29	0.77
Antibiotic use	–	–	8.73	<0.01^**^
Yes	57	33		
No	33	48		
Gesell				
Adaption (DQ)	65.96 ± 15.62	98.54 ± 12.14	−15.63	0.00^***^
Gross motor (DQ)	73.17 ± 13.41	99.84 ± 12.03	−14.07	0.00^***^
Fine motor (DQ)	72.12 ± 16.95	98.72 ± 12.20	−12.09	0.00^***^
Language (DQ)	53.71 ± 20.85	103.94 ± 15.95	−18.16	0.00^***^
Personal-social (DQ)	56.96 ± 15.32	106.52 ± 20.19	−18.64	0.00^***^
CARS	37.30 ± 4.04	–	–	
ABC	53.28 ± 22.28	–	–	
ADI-R				
Social Interaction	19.26 ± 4.69	–	–	
Stereotyped behavior	6.23 ± 2.23	–	–	
High-level stereotyped behavior	3.04 ± 1.66	–	–	
Low-level stereotyped behavior	2.88 ± 1.1.3	–	–	
Disease development	3.81 ± 0.90	–	-	
ADOS		–	–	
Social Interaction	11.39 ± 1.53	–	–	
Communication	6.54 ± 1.49	–	–	
Combined	17.81 ± 2.66	–	–	
Imagination/creativity	2.68 ± 1.04	–	–	
Stereotyped behave/Restricted interests	3.80 ± 1.15	–	–	

ASD, autism spectrum disorder; TD, typical development; BMI, body mass index; Gesell, Gesell developmental schedules; CARS, childhood autism rating scale; ABC, autism behavior checklist; ADI-R, autism diagnostic interview-revised; ADOS, autism diagnostic observation scale;

* P < 0.05;

** *P < 0.01*,

*** *P < 0.001*.

### Comparison of the Incidence of Aberrant EBs and GI Symptoms Between the ASD Group and the TD Group

**Figure 1:**
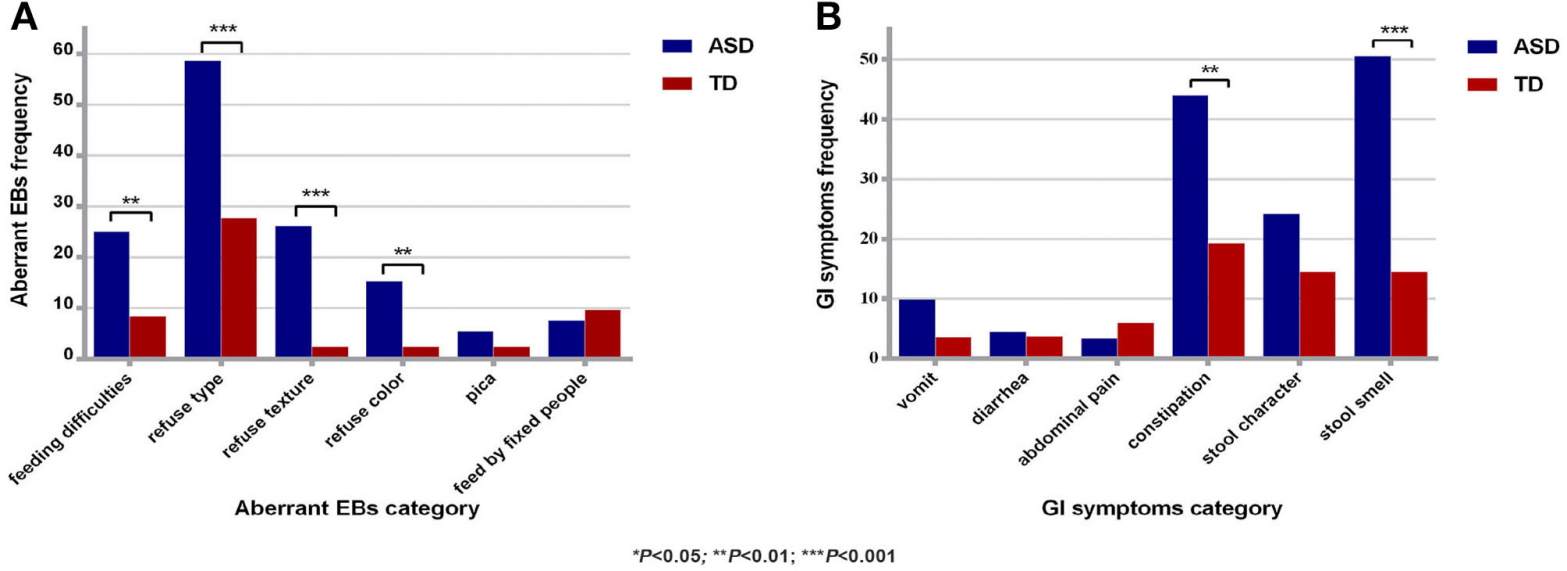
Comparison of aberrant EBs and GI symptoms between the two groups. **(A)** Comparison of aberrant EBs between the two groups. **(B)** Comparison of GI symptoms between the two groups. ASD, autism spectrum disorder; TD, typical development; aberrant EBs, aberrant eating behaviors; GI symptoms, gastrointestinal symptoms; *P < 0.05; **P < 0.01, ***P < 0.001.

There were 91 and 76 valid questionnaires regarding aberrant EBs and GI symptoms for the ASD group and the TD group, respectively. The effective response rate for the questionnaire was 90.76%. The abnormal EB detection rate for the ASD group was significantly higher than that for the TD group (67.39 and 34.94%, respectively); the difference was significant (χ^2^ = 18.41, *P* < 0.01). The average score for EBs in the ASD group (1.38 ± 1.36) was significantly higher than that in the TD group (0.53 ± 0.89) (*t* = 4.85, *P* < 0.01). Child refuses food based on type, refuses food based on texture, and feeding difficulties were the top 3 EBs in the ASD group. The detection rates of GI symptoms in the ASD group and the TD group were 80.22 and 42.11%, respectively; the difference was significant (χ^2^ = 25.77, *P* < 0.01). The average score for GI symptoms (1.49 ± 1.21) in the ASD group was significantly higher than that in the TD group (0.71 ± 0.99) (*t* = 4.53, *P* < 0.01). Stool smell, constipation, and abnormal stool character were the top 3 GI symptoms in the ASD group (Figure 1).

### Food Intolerance and Specific IgG Antibody Concentrations in the ASD Group

In this study, a total of 89 children with ASD received testing for 14 kinds of food intolerance. The Specific IgG antibody concentrations and levels distribution of food intolerance are shown in Table 2. The positive rate for total items of food intolerance in children with ASD was 17.74%, and the average concentration of specific IgG antibodies for every child was (448.17 ± 284.29) U/ml. The results showed that 89.89% (80 children) of children with ASD had at least one food intolerance item, of which children with 6 food intolerance items accounted for 4.49% (4 children), children with 5 food intolerance items accounted for 2.25% (2 children), children with 4 food intolerance items accounted for 16.85% (15 children), children with 3 food intolerance items accounted for 22.71% (22 children), children with 2 food intolerance items accounted for 22.71% (22 children), and children with only one food intolerance item accounted for 18.00% (16 children). Eggs, milk, and wheat were the top 3 food intolerance items for children with ASD, and the positive rates were 84.27, 65.17, and 38.20%, respectively.

**Table 2:** Specific IgG antibody concentrations and levels distribution of food intolerance in the ASD group.

	**Levels distribution (cases)**				**Concentrations ($\bar{\text{x}}$ ± *S*)**
	**–**	**+**	**++**	**+++**	
Rice	86	1	1	1	16.76 ± 28.82
Milk	31	22	25	11	102.83 ± 111.84
Egg	14	14	33	28	165.26 ± 104.80
Wheat	55	21	13	0	48.57 ± 46.46
Soybean	75	11	3	0	24.88 ± 31.31
Tomato	79	9	1	0	28.54 ± 28.44
Beef	87	2	0	0	7.24 ± 11.92
Chicken	87	1	1	0	13.99 ± 21.73
Pork	89	0	0	0	5.94 ± 5.89
Crab	88	0	1	0	11.37 ± 28.10
Shrimp	88	0	1	0	8.39 ± 15.37
Codfish	70	14	5	0	29.74 ± 40.44
Mushroom	89	0	0	0	6.34 ± 7.16
Corn	87	1	1	0	14.37 ± 18.29
Total	–	–	–	–	448.17 ± 284.29

***–**, negative (IgG <50 U/ml); +, mild intolerance (IgG: 50~100 U/ml); ++, moderate intolerance (IgG: 100~200 U/ml); + + +, severe intolerance (IgG>200 U/ml)*.

### Analysis of the Correlation Among Clinical Core Symptoms and Aberrant EBs and GI Symptoms and Specific IgG Antibody Concentrations in the ASD Group

The results of pairwise correlation analysis between scores of all scales of clinical core symptoms in children with ASD and scores for aberrant EBs and GI symptoms as well as specific IgG antibody concentrations are shown in Table 3. Aberrant EBs score with stereotyped behavior score of the ADI-R had a positive correlation(*r* = 0.21, *P* = 0.04), with the total ABC scale score, the scores for the B, S, and SC factors also had a positive correlation (all *P* < 0.05). The average concentration of specific IgG antibodies with the score for high-level stereotyped behavior score on the ADI-R scale showed a positive correlation (*r* = 0.23, *P* = 0.03); there were no other significant correlations found (all *P* > 0.05).

**Table 3:** Correlation between aberrant EBs, GI symptoms, average IgG antibody concentrations of every participant and clinical core symptoms.

	**aberrant EBs**		**GI symptoms**		**IgG antibody**	
	***r***	***P***	***r***	***P***	***r***	***P***
ADI-R						
Social interaction	0.09	0.42	**–**0.05	0.67	**–**0.01	0.97
Stereotyped behavior	0.21	0.04^*^	0.07	0.51	0.05	0.63
High-level stereotyped behavior	0.11	0.32	0.05	0.65	0.23	0.03^*^
Low-level stereotyped behavior	0.20	0.06	**–**0.01	0.97	**–**0.10	0.35
Disease development	0.10	0.34	0.08	0.44	0.05	0.61
ADOS						
Social interaction	0.01	0.91	0.15	0.15	**–**0.19	0.06
Communication	**–**0.03	0.75	0.11	0.30	**–**0.10	0.33
Combined	**–**0.00	0.99	0.11	0.32	**–**0.17	0.11
Imagination/creativity	**–**0.08	0.44	0.18	0.10	**–**0.15	0.15
Stereotyped behave/Restricted interests	0.13	0.24	0.12	0.24	**–**0.17	0.10
CARS	0.11	0.29	0.09	0.37	**–**0.15	0.15
ABC	0.35	0.00^**^	0.12	0.27	**–**0.10	0.35
Body and object use	0.33	0.00^**^	0.12	0.28	**–**0.11	0.34
Language	0.19	0.08	0.06	0.60	**–**0.11	0.30
Sensory	0.30	0.00^**^	0.09	0.40	**–**0.05	0.62
Relating	0.20	0.07	0.06	0.61	**–**0.11	0.34
Social and self-help	0.29	0.01^**^	0.14	0.20	**–**0.09	0.42
aberrant EBs	–	–	**–**0.10	0.33	**–**0.10	0.36
GI symptoms	–	–	–	–	**–**0.08	0.45
IgG antibody	–	–	–	–	–	–

aberrant EBs, aberrant eating behaviors; GI symptoms, gastrointestinal symptoms; ADI-R, autism diagnostic interview-revised; ADOS, autism diagnostic observation scale; CARS, childhood autism rating scale; ABC, autism behavior checklist;

* P < 0.05;

** *P < 0.01*,

***P < 0.001.

## Discussion

Aberrant EBs and GI symptoms are common accompanying problems in children with ASD and place enormous pressure on parents and families. Currently, the association between these accompanying behaviors and ASD clinical core symptoms as well as whether they correlate with the pathogenesis of ASD are still not clear. Therefore, an investigation of aberrant EBs and GI symptoms in children with ASD can not only provide a better understanding of behavioral characteristics of this population but probably provide an evidence for understanding underlying pathogenesis of its development and of interventions and treatments.

This study showed that the detection rate of aberrant EBs in preschool children with ASD was 67.39% and was significantly higher than that in TD children; in addition, aberrant EBs were concentrated in sensory perceptions associated with food selection: colors, textures, and types. Three Chinese surveys on aberrant EBs among children with ASD aged between 2 and 7 years from Heilongjiang (13), Qingdao (14), and Xinjiang (15) showed that the detection rate was between 76.6 and 89.8%, which was similar to the result in our study. The differences in the detection rates among similar studies in other countries were large. These differences might due to the following reasons. First, the evaluation methods of symptoms were different. Currently, the majority of questionnaires are filled out by parents, but some questionnaires are completed by clinical physicians. Next, the evaluation tools were different. For GI symptoms or aberrant EBs, some researchers used the Autism Treatment Evaluation Checklist (ATEC), and some researchers used self-compiled questionnaires (4, 16); therefore, the evaluation criteria of symptoms were different, and the surveyed age periods were also different (2). In our study, the GI symptoms and aberrant EBs were surveyed by self-compiled questionnaire, which was prepared based on clinical observation and previous studies, furthermore, the questionnaires filled out by the parents were checked by the researchers and the parents together to ensure that the information filled in is true and reliable.

For the association between EBs and ASD clinical core symptoms, the results of a study by Japanese scholar Miho Shizawa showed that the number of aberrant EBs were associated with the development of ASD and that the severity of sensory sensitivity was positively correlated with the presence of aberrant EBs (17). It is currently considered that abnormal perception is one of the ASD clinical symptoms. The majority of children with ASD will exhibit abnormal sensory perception regarding, for example, smell, taste, and texture (18), and several neurophysiological studies assumed that the clinical sensory problems are results of perceptual deficits in processing tactile information (19). Therefore, the authors of that study proposed that abnormal perception might be a reason for aberrant EBs such as food selectivity and pica in children with ASD. Our study further investigated the correlation between aberrant EBs and ASD clinical core symptoms. The results showed that aberrant EBs scores were positively correlated with stereotyped behaviors in ASD. This is not difficult to understand, because according to clinical observation, the stereotypical behavior of some ASD children is reflected in the eating behavior. The study by Prosperi in Italy also showed that preschool children with ASD with accompanying aberrant EBs had more severe stereotyped behaviors and restricted interests than did those without aberrant EBs (4), which is consistent with our study. A study by Suarez in the USA on school children with ASD showed that aberrant EBs of children with ASD was associated with both their stereotyped behaviors and sensory over-responsivity (20). The combination of existing results showed that EBs in ASD not only is a very common accompanying problem but also requires clinical attention; in addition, EB symptoms are closely associated with stereotyped behaviors and abnormal sensations of ASD. Aberrant EBs are complex issues of ASD. Riccio's research proved that aberrant EBs, in particular food selectivity was mediated by taste sensitivity which related to TAS2R38 genotype (21). Messina's study presented that the rule of sympathetic activity was very important for ASD, neurotransmitters alterations could explain food selectivity (22), and further study could broaden horizons to multi-modal approach.

Constipation, diarrhea, and vomit are intestinal signs and symptoms. This study also surveyed GI symptoms in preschool children with ASD. The results showed that the detection rate of GI symptoms in children with ASD reached 80.22% and was significantly higher than that in TD children. The prevalence of GI symptoms suggests a possible association between gut microbiota and ASD (23). In addition, the study in preschool children reported that ASD had unbalance gut microbiota structure (24). Further correlation analysis in our study did not show a correlation between GI symptoms and ASD clinical core symptoms, whereas a study by Yang of 169 preschool children with ASD showed that the severity of GI symptoms was positively correlated with the severity of ASD clinical core symptoms (5). A study by Chandler of 132 children with ASD aged between 10 and 14 years did not show that correlation (6). In addition, the conclusions in a study by Mazefsky et al. (25) of 95 patients with ASD aged between 7 and 19 years were similar to those of our study. Children suffer from GI problems usually have the problem of GI functions, while except for GI dysfunction, the organ changes, endocrine stress responses, imbalance of gut microbiota, activation of the sympathetic nervous system, and the hypothalamic-pituitary axis were also proved associated with GI problems in ASD and should be considered (5, 26).

As a type III allergic reaction, food intolerance is more common among the entire child population. Li in China reported that the percentage of children with ASD aged between 2 and 7 years with food intolerance was significantly higher than that in children at the same age (27). Our study showed that the majority of children with ASD had food intolerance. The most common food intolerance items were eggs, milk, and wheat. This result is consistent with the results of many domestic and international studies (27–30). The mechanism underlying food intolerance is still not clear. Saad considered that the reason of food intolerance might be a lack of corresponding proteases in the body (31); therefore, food proteins cannot be completely digested and absorbed and thus recognized as antigens in the body, inducing allergic reactions. Some researchers also believe that food intolerance is associated with microbiota-gut brain (MGB) axis, an impaired intestinal mucosal barrier. Children with ASD are usually reported to have a “leaky gut” (32), altered gut permeability play a pathogenic role allowing the input of molecules, the gut microbiota can act through the permeability of gut barrier, undigested proteins are also absorbed by the body through an incomplete intestinal mucosal barrier and pass through the blood–brain barrier to activate the immune system in an abnormal way (23). As in the previous mentioned, food compounds combine with food IgG antibodies to form immune complexes, and triggers immune-inflammatory cascade, which may contribute to low-level systemic inflammation and low-level neuroinflammation, via pathological processes in CNS (7), like increased expression of Kynurenine (33), and the inflammatory molecules interleukin (IL-1β), IL-6, IL-17, and tumor necrosis factor (TNF) in the brain (34). Many neurochemical pathways also involved in ASD, for instance, gamma aminobutyric acid (GABA), glutamate, serotonin, dopamine, oxytocin, vitamin D, Marotta et al. (35). In conclusion, various studies have suggested that food intolerance is related to immunity, and previous studies have also shown that there are immune system abnormalities in part of ASD (36). Our study didn't find significant results between concentrations of specific IgG antibodies and GI symptoms. A few of previous studies proved the relationship between these. It's difficult to say the serum specific IgG antibodies are the cause or results of GI symptoms. On the one hand, increased gut permeability could potentially result in elevated levels of the food antibodies, while there was an inverse association between tight-junctional gut permeability and perceived food intolerance (37), suggesting other underlying mechanism for the concentrations of antibodies. On the other hand, GI symptoms are only a clinical manifestation, which could be caused by digestive disease, intestinal flora disturbance, or even organic disease. However, the causal relationship between food-specific IgG antibodies and GI symptoms remains unclear, further research is needed, especially at related cytokines levels.

On the basis of the detection of concentration of food-specific antibodies in children with ASD, this study further investigated the correlation between food-specific IgG antibody concentration and aberrant EBs, GI symptoms as well as ASD clinical core symptoms. The results showed that food-specific IgG antibody concentration correlated with high-level stereotyped behavior of ASD, indicating that when specific IgG concentration in children with ASD was higher, the high-level stereotyped behavior was more severe. More convincing evidence comes from Martin et al., the research injected IgG antibodies from mothers of children with ASD and IgG antibodies from mothers of TD children into pregnant rhesus monkeys (38). The infant rhesus monkeys presented more atypical repetitive behaviors after being injected with IgG antibodies from mothers of children with ASD. Singer et al. (39) and Camacho et al. (40), used pregnant mice to perform similar studies and showed that neonatal mice had more stereotyped behaviors and social problems after being injected with antibodies from mothers of children with ASD. More recent study provided evidence of abnormalities in brain volume of IgG-ASD children and rhesus monkeys (41, 42). Cytokines regulate brain activity through the brain's gut axis, for instance, high-level brain-derived neurotrophic factor (BDNF), and low-level tryptophan and kynurenic acid were characteristics of ASD, abnormal EEG results were associated with an elevated BDNF/PRO-BDNF ratio (43). The elevation of IL-6 in the brain could involve in the mediation of autism-like behaviors through impairments of neuroanatomical structures and neuronal plasticity (44). Dendritic cells are major players in innate immunity and are import in activation of naïve T cells, induction of tolerance and cytokine/chemokine production. A more compelling study suggest the frequencies of myeloid dendritic cells were significantly increased in children with ASD, elevated frequencies of myeloid dendritic cells were positively associated with amygdala volume, GI symptoms and stereotyped behaviors (45). These studies all provide some evidence for the involvement of brain-gut-axis mechanisms in the pathogenesis of ASD. The results in this study showed that stereotyped behaviors of preschool children with ASD not only correlated with aberrant EBs symptoms but also correlated with food-specific IgG antibody levels, which may have implications for exploring the immune mechanism of ASD. These studies implicate the role of immune system abnormality in the development and progression of ASD is quite important and worthy of in-depth studies. Further study we will focus on cytokines levels, and explore the underlying mechanism of related symptoms. This study had some limitations. First, aberrant EBs and GI symptoms involved in this study were acquired through a questionnaire; therefore, there was a certain degree of subjectivity. Second, although the significant correlation among specific IgG antibodies, aberrant EBs and the clinical symptoms, the correlation coefficients in the study are not high, which may be related to the insufficient sample size. In future studies, we can increase the sample size and investigate children with ASD at older ages.

## Data Availability Statement

The datasets generated for this study are available on request to the corresponding author.

## Ethics Statement

The studies involving human participants were reviewed and approved by the Medical Ethics Committee of the Brain Hospital Affiliated with Nanjing Medical University (approval number: 2016–KY017). Written informed consent to participate in this study was provided by the participants' legal guardian/next of kin.

## Author Contributions

CL and XK contributed to conception and design of the study. CL wrote the first draft of the manuscript. All authors performed the data collection and analyses and have approved the final manuscript.

## Conflict of Interest

The authors declare that the research was conducted in the absence of any commercial or financial relationships that could be construed as a potential conflict of interest.
